# Green-synthesized zinc oxide nanoparticles using *Echinops spinosus* extract: A promising anticancer agent for hepatocellular carcinoma

**DOI:** 10.1371/journal.pone.0331171

**Published:** 2025-10-23

**Authors:** Rabab S. Hamad, Mohammed Ahmed Ali Elshaer, Hesham A. El-Mahdy, Osama A. Mohammed, Mohammed Jeelani, Ahmed S. Doghish, Mohamed Awad Mahmoud Abd-Elraheem, Mervat Mostafa Abdel mageed, Amira Abd-elfattah Darwish, Elwathiq Khalid Ibrahim, Walaa A. El-Dakroury

**Affiliations:** 1 Biological Sciences Department, College of Science, King Faisal University, Al Ahsa, Saudi Arabia; 2 Agricultural Biochemistry Department, Faculty of Agriculture, Al-Azhar University, Cairo, Egypt; 3 Biochemistry and Molecular Biology Department, Faculty of Pharmacy (Boys), Al-Azhar University, Nasr City, Cairo, Egypt; 4 Department of Pharmacology, College of Medicine, University of Bisha, Bisha, Saudi Arabia; 5 Department of Physiology, College of Medicine, University of Bisha, Bisha, Saudi Arabia; 6 Department of Biochemistry, Faculty of Pharmacy, Badr University in Cairo (BUC), Badr City, Cairo, Egypt; 7 Medical Physics Basic Science Department, Faculty of Physical Therapy, Egyptian Chinese University, Egypt; 8 Department of Medical Laboratory Technology, Faculty of Applied Health Sciences Technology, Pharos University in Alexandria, Alexandria, Egypt; 9 Department of Anatomy, College of Medicine, University of Bisha, Bisha, Saudi Arabia; 10 Department of Pharmaceutics and Industrial Pharmacy, Faculty of Pharmacy, Badr University in Cairo (BUC), Badr City, Cairo, Egypt; Yakin Dogu Universitesi, TÜRKIYE

## Abstract

The environmentally sustainable synthesis of nanoparticles has arisen as a viable alternative to traditional methods, tackling ecological and economic issues. This research investigates the green synthesis of ZnO nanoparticles utilizing an aqueous extract of *Echinops Spinosus* L. roots (ESRE), abundant in bioactive chemicals, as a natural reducing agent. The impacts of different quantities of precursors and reducing agents were methodically examined. The synthesized ZnO nanoparticles were characterized by zeta potential measurements (−15.2 mV), transmission electron microscopy (TEM), which indicated spherical and hexagonal morphologies with an average size of 20.47–48.22 nm, and energy-dispersive X-ray spectroscopy (EDX), confirming zinc and oxygen as the principal elements. Fourier transform infrared spectroscopy (FTIR) underscored plant-derived compounds’ contribution to nanoparticle stabilization. Cytotoxicity was assessed using the MTT assay on two cancer cell lines, HepG2 (hepatocellular carcinoma) and MCF-7 (breast cancer). The green-synthesized ZnO nanoparticles had substantial anticancer efficacy, with the ZnO nanoparticles exhibiting the most pronounced anti-proliferative effect on HepG2 cells (10.4 folds) and 5.4 folds more effective in MCF-7 cells compared to ESRE, with HepG2 cells IC50 value of 19.94 ± 0.11 µg/mL while the IC50 for MCF-7 cells was 75.65 ± 0.16 µg/mL. The results indicate that ZnO-NPs produced by green technologies exhibit significant potential as anticancer agents, especially for hepatocellular carcinoma treatment.

## 1. Introduction

Cancer continues to be a predominant cause of mortality globally, and although traditional therapies like chemotherapy, radiation, and immunotherapy have progressed considerably, they still encounter severe challenges that restrict their efficacy and safety. Conventional chemotherapeutic drugs are frequently non-specific, affecting both malignant and healthy cells, resulting in significant adverse effects and constraining the safely permissible dosage [1]. Tumors often acquire resistance to various medications, typically by mechanisms like the P-glycoprotein (P-gp) drug efflux pathway, which aggressively removes pharmaceuticals from cancer cells, hence lowering intracellular drug levels and impairing treatment effectiveness. Moreover, the compact extracellular matrix and aberrant vasculature of tumors impede medication infiltration, limiting therapeutic effectiveness in solid tumors [2].

Nanoparticles have emerged as a revolutionary method in cancer therapy, overcoming significant limits inherent in traditional treatments such as chemotherapy, radiation, and immunotherapy. The principal advantage of nanoparticles is their capacity to improve drug delivery via size- and surface-modifiable characteristics, facilitating precise and regulated medicinal administration [3].

Engineered nanoparticles can exploit the distinctive tumor microenvironment, particularly the enhanced permeation and retention (EPR) effect, to preferentially accumulate in tumor tissues while preserving healthy cells. This targeting capability enhances the efficiency of anticancer medicines while diminishing systemic toxicity, a significant issue associated with conventional therapies that lack specificity [4]. Besides targeting and improved penetration, nanoparticles provide solutions to the issue of multi-drug resistance (MDR), a major obstacle in cancer treatment. Cancer cells frequently develop medication resistance by overexpressing drug-efflux pumps P-glycoprotein (P-gp), which actively extrude therapeutic drugs from the cells. Nanoparticle-based delivery devices can circumvent these efflux processes, facilitating elevated intracellular drug concentrations and enhancing treatment efficiency in resistant cancer cell types [5].

Metallic nanoparticles (MNPs) evolved as exceptionally promising agents in cancer therapy, providing distinct advantages that conventional treatments find challenging to mimic. Because of their diminutive dimensions, generally within the nanometer scale, MNPs can effectively infiltrate tumors and engage at the cellular level, facilitating accurate targeting and regulated release of therapeutic drugs. One of the primary advantages of metallic nanoparticles is their capacity to facilitate selective drug delivery to cancer cells while preserving healthy tissue, thus limiting off-target effects and decreasing systemic toxicity [6]. A significant benefit of MNPs is their ability to address MDR. By circumventing cellular efflux pumps and promoting increased drug retention in cancer cells, MNPs augment medication efficacy in instances where traditional treatments falter due to resistance mechanisms [7].

Among various types of MNPs, Zinc oxide (ZnO) nanoparticles are increasingly recognized for their unique physicochemical and biological characteristics in anticancer applications. ZnO nanoparticles exhibit significant biocompatibility and can be manufactured through environmentally friendly ways, utilizing plant extracts that augment their medicinal efficacy. These nanoparticles demonstrate potent cytotoxicity towards cancer cells, mainly by generating reactive oxygen species (ROS) that create oxidative stress and subsequently trigger death in cancer cells while minimally affecting healthy cells [8]. Moreover, ZnO nanoparticles produced using plant extracts have enhanced stability and tailored efficacy, facilitating selective accumulation in tumor tissues. Plants exhibit substantial genetic diversity concerning the quantity of biomolecules and metabolites, including proteins, vitamins, coenzyme-based intermediates, phenols, flavonoids, and carbohydrates. These plant metabolites possess hydroxyl, carbonyl, and amine functional groups that interact with metal ions and diminish their size to the nanoscale. Flavonoids possess multiple functional groups, and it is posited that the -OH group is primarily accountable for reducing the metallic ions into nanoparticles. These compounds facilitate the bioreduction of ions to nanoscale dimensions and are crucial for the capping of nanoparticles, essential for durability and biocompatibility. Reducing agents, including phenolic chemicals, sterols, and alkaloids, can convert metal ions into nanoparticles in a single process [9,10]. ZnO nanoparticles obtained from *Aquilegia pubiflora* demonstrated significant toxicity to liver cancer cells through the induction of reactive oxygen species, emphasizing their efficacy for targeted cancer therapy [10].

ZnO has been identified as potentially more profitable and effective than other metals for the biosynthesis of nanoparticles for clinical applications. Numerous investigations have proven the manufacture of ZnO nanoparticles utilizing various plant extracts. For instance, the floral extract of the medicinal herb *Cassia auriculata* and the leaf extract of *Hibiscus rosasinensis* were employed as reducing agents for zinc nitrate to synthesize ZnO nanoparticles [9].

*Echinops spinosus (ES)*, a member of the Asteraceae family, consists of roughly 120 species distributed over the Mediterranean, Central Asia, and tropical Africa. The species *spinosus* exhibits vigorous development in arid regions with precipitation levels between 20 and 100 mm and shows adaptation to several soil types, thriving in coastal dunes as well as sandy, gravelly, and rocky landscapes [11]. The geographic distribution of ES encompasses the Sahara region, including Sinai and the Red Sea coast, signifying a prevalent occurrence. ES is acknowledged as one of the five species associated with this genus throughout Egypt [12]. ES was extensively utilized in traditional medicine for enhancing blood circulation, managing diabetes, addressing spasmolytic issues, alleviating gastrointestinal pain, treating indigestion, and functioning as an abortifacient and diuretic. Furthermore, it is a therapeutic herb with curative characteristics, including antimicrobial, antioxidant, and anti-inflammatory effects [13–16]. The unique medicinal properties of ES can be ascribed to its composition of sesquiterpenes, quinoline alkaloids, phenols, and flavonoids [17].

Compared with recent related studies, our work uniquely focuses on the green synthesis of ZnO NPs using *Echinops spinosus* root extract, which has not been previously reported. While other studies mainly explored antimicrobial [18,19], tissue imaging [20] or bone regeneration effects [21]. Our study emphasizes anticancer activity with detailed IC50-based cytotoxicity on HepG2 and MCF-7 cells, along with phytochemical profiling and nanoparticle characterization. This integrated approach enhances the relevance of our findings for early-stage cancer nanotherapeutics.

We present a straightforward and environmentally eco-friendly approach for synthesizing ZnO nanoparticles using plant extracts from ES as reducing agents and zinc acetate as a precursor, aimed at a comparative investigation of their anticancer efficacy against liver and breast cancer. This discovery will enhance the applicability of plant-based nanoparticles in the biomedical industry, particularly in cancer therapy.

## 2. Materials and methods

### 2.1 Chemicals and reagents

The 3-(4,5-Dimethyl-2-thiazolyl)-2,5-diphenyl-2H-tetrazolium-bromide (MTT), Zinc acetate, sodium hydroxide, and dimethyl sulfoxide (DMSO) were purchased from Sigma Chemical co (St. Louis, Missouri, USA), fetal bovine serum (FBS), phosphate buffer saline (PBS), Dulbecco’s modified Eagle’s medium (DMEM), penicillin/streptomycin (Pen/Strep) solution and trypsin- EDTA were procured from Gibco (Gibco, TFS Inc., USA).

### 2.2 Plant materials and preparation of extract

The ESRE was prepared according to a previously reported method with some amendments [13,14]. Fresh roots of ES were harvested from the location 47 km along the Alexandrian coast, near Borg Elarb, Alexandria, Egypt (30°56’52.0“N 29°31’10.5” E). Subsequently, it underwent multiple washing cycles with distilled water (D.W.) to remove any dust particles and pollutants on its roots. Afterward, the roots were air-dried, fragmented into the smallest fragments, and subjected to extraction by the decoction method using D. W. Throughout the extraction process, 100 g of ES roots was utilized in 1000 mL of D. W. at 75 °C for 2 h with constant stirring. The extract was first passed through muslin cloth and then underwent three filtrations using Whatman filter paper No. 1. The ultimate solution of ESRE was preserved at 4°C for future assessment and utilization.

### 2.3 Phytochemical scanning

Phytochemical analyses have been extensively scrutinized to assess and characterize bioactive chemical compounds in aqueous ESRE, as delineated in contemporary literature [22–24].

### 2.4 HPLC analysis

An Agilent 1260 infinity series (CA, USA) instrument was utilized for HPLC analysis. The chromatographic process employed a C18 column, specifically the Eclipse model (4.6 mm x 250 mm i.d., 5 μm). The mobile phase consisted of 0.05% trifluoroacetic acid in acetonitrile (B) and water (A) at a flow rate of 0.9 ml/min. A gradient elution protocol was executed with exacting composition and timing: 0 minutes (82% A), 0–5 minutes (80% A), 5–8 minutes (60% A), 8–12 minutes (60% A), 12–15 minutes (82% A), 15–16 minutes (82% A), and 16–20 minutes (82% A). Detection was performed using a multi-wavelength detector calibrated to 280 nm, with an injection volume of five microliters for each sample solution [25,26]. The column temperature was meticulously maintained at 40 °C.

### 2.5 Preparation of green zinc oxide nanoparticles

Zinc oxide nanoparticles were obtained by sustainable processes utilizing the precipitation technique, which is recognized for its ecologically friendly characteristics [27]. Zinc acetate was employed as a Zn precursor in the production of nanoparticles, where zinc acetate undergoes hydrolysis to yield acetate and zinc ions upon heating. A 0.01 M solution of Zn acetate was precisely produced, subsequently, 10 mL of ESRE for every 100 mL of the zinc acetate solution was incorporated. The ESRE extract served as a reducing, capping, and stabilizing agent for nanoparticle production, thus eliminating the use of environmentally deleterious substances. The resultant reaction admixture was subjected to stirring at 300 rpm and 75 °C for 2 h. The pH of the reaction was meticulously controlled by the stepwise inclusion of 1 M NaOH at a steady rate until a pH of 12. An observable change was observed in the solution, triggering the formation of a white precipitate. Upon completion of the reaction, the mixture underwent centrifugation for 30 min at 4000 rpm to separate the supernatant from the precipitate, which was thereafter collected, dried, and subjected to annealing in an oven at 400 °C for 2 h.

### 2.6 Characterization of ZnO nanoparticles

The synthesized ZnO nanoparticles (NPs) capped with aqueous ESRE underwent analysis utilizing Zeta potential, Transmission Electron Microscope (TEM), and Energy Dispersive Analysis of X-ray (EDX). Zeta potential measurements were executed to examine the electrical charge and ascertain the stability of the green ZnO-NPs, employing a zeta voltage test on a Zeta sizer (ZS) from Malvern Instruments Inc., Malvern. UK, following the previously reported procedure [28]. Transmission Electron Microscope (JOEL, JEM-1400, Tokyo, Japan) was utilized for particle size and morphology analysis. The freshly prepared sample was diluted just before the microscopy procedure. A copper grid was subsequently dipped in the sample multiple times before being stained with uranyl acetate. Upon drying the samples, they were subsequently placed and scanned at 70kV. Subsequent evaluation of the images was performed using ImageJ software on a GEOL [29]. Verification of elemental presence was achieved through Energy Dispersive Analysis of X-ray (EDX). The microscopic EDX analysis was conducted utilizing an X-ray precision analyzer (Oxford 6587 INCA) connected to a JEOL JSM-5500 LV electron microscope operating at 20 kV [30,31].

### 2.7 FTIR analysis

The Fourier transform infrared (FTIR) spectrum was determined using a JASCO FT-IR 3600 model spectrophotometer with KBr pellets, the spectral information was gathered across the wave number range of 400–4000 cm^-1^ to detect the functional groups in aqueous ESRE and green synthesized ZnO NPs [32]. The sample was pulverized with KBr and formed into a disk of appropriate size (13 mm) for assessment [33,34].

### 2.8 Anticancer activity

#### 2.8.1 Cell lines.

The breast cancer cell line (MCF-7) and liver cancer cell line (HepG2) were purchased from ATCC (Manassas, USA) and cultured in DMEM (Invitrogen) supplemented with 10% fetal bovine serum (FBS) and 1% penicillin/streptomycin solution (TFS Inc., USA) at a temperature of 37 ˚C with 5% carbon dioxide. This study didn’t involve any animal or human experiments. The BUC University Research Ethics Committee has confirmed that no ethical approval is required.

#### 2.8.2 Cell viability assay.

The cytotoxic capacity was quantified using the MTT technique [35]. The cells were seeded in 96-well plates at an average density of 1.2 × 10^4^ cells per well and allowed to develop for 24 h. Then, the medium containing different concentrations of nanoparticles and extract was replenished. The MTT assay occurred after 48 h by introducing 100 μL of a solution comprising 5 mg/mL of MTT in PBS. The wells were then incubated at 37°C for 4 h. 100 μL of DMSO was applied to every well to solvate the formazan crystals. The plates were incubated at 37°C for 10 min. Optical densities have been estimated using measurements at 570 nm obtained from a Microplate reader (Epoc-2 C microplate reader, Bio Tek, USA).

### 2.9 Statistical analysis

The GraphPad Prism 8.0 (San Diego, CA) was employed to conduct data analysis and graphical demonstrations. All results were given as Means ± SD, and all experiments were done in triplicate (n = 3). The statistical analysis was conducted using one-way analysis of variance (ANOVA) and Tukey’s multiple comparison tests. P < 0.05 was significant.

## 3. Results and discussion

### 3.1 Phytochemical screening of aqueous (ESRE)

The findings of a qualitative analysis on the identification of bioactive compounds, including flavonoids, phenols, saponins, tannins, and alkaloids in the crude aqueous solution of (ESRE. The results indicated that the aqueous (ESRE) encompassed all listed components, suggesting a constant presence of these compounds [14,36].

In the green synthesis of metal nanoparticles, these plant metabolites have been reported as reducing and stabilizing agents; they also serve as antioxidants [37].

### 3.2 HPLC analysis

**Fig 1:**
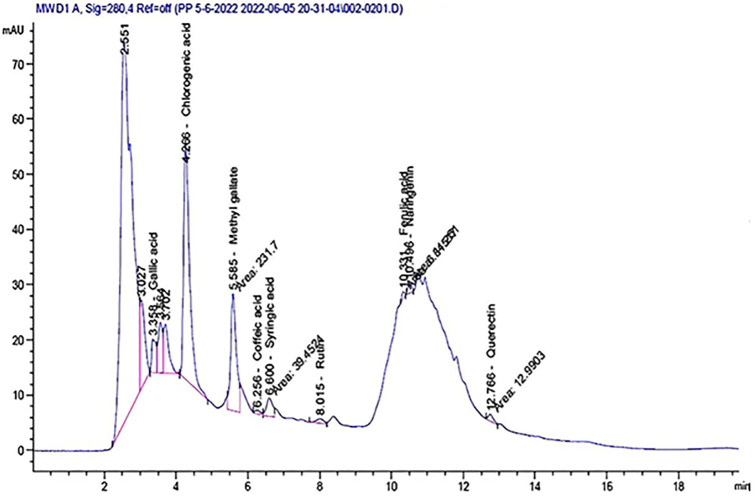
HPLC chromatogram of aqueous ESRE.

The HPLC assessment of the aqueous extract (ESRE) of the plant demonstrated a wide array of phenolic compounds at varying concentrations, signifying a rich phytochemical profile. To obtain a more thorough depiction of the polyphenolic compounds present in aqueous (ESRE), a direct and effective analytical approach employing a mobile phase composed of water and trifluoroacetic acid in acetonitrile was developed through the application of High-Performance Liquid Chromatography (HPLC). Phenolic compounds specified within the aqueous (ESRE) are delineated in Table 1 and Fig 1, showcasing distinct peaks corresponding to varying retention times (RT). Out of the total of compounds detected, 9 poly phenolic components were observed, among which Chlorogenic acid exhibited the highest concentration of 75 µg/mL followed by Methyl gallate 13.81 µg/mL, then, Gallic acid, Syringic acid, Quercetin, Rutin, Naringenin, Coffeic acid and Ferulic acid recorded 4.36, 3.73, 1.55, 1.51, 1.24, 0.53 and 0.43 µg/mL respectively. A similar finding was reported [17,38,39]

**Table 1:** HPLC analysis of aqueous (ESRE).

Sample ECH (1g/10 ml)	Area	Conc. (µg/ml)	Conc. (µg/g)
Gallic acid	51.95	4.36	43.60
Chlorogenic acid	502.18	75.00	750.04
Methyl gallate	231.70	13.81	138.14
Coffeic acid	6.97	0.53	5.28
Syringic acid	39.45	3.73	37.33
Rutin	11.74	1.51	15.05
Ferulic acid	6.82	0.43	4.28
Naringenin	14.25	1.24	12.39
Quercetin	12.99	1.55	15.52

Polyphenolic substances, particularly phenolic acids and flavonoids, are recognized for their antioxidant properties and have garnered significant interest over time. The identification of these chemicals is steadily increasing due to their advantageous impacts on health and disease [40–42].

Chlorogenic acid, the predominant ingredient in the extract, is well-established for its antiproliferative effects on several cancer cells. Research indicates that chlorogenic acid can trigger apoptosis by interfering with cell cycle progression, suppressing the NF-κB pathway, and elevating oxidative stress in cancer cells, potentially affecting both Hep-G2 and MCF-7 cells significantly. This mechanism corresponds with the effects found in the present investigation, indicating that chlorogenic acid may significantly contribute to the extract’s anticancer efficacy [43,44].

Methyl gallate and gallic acid, present in significant quantities, are recognized for their capacity to generate oxidative stress in cancer cells, potentially resulting in apoptosis and the inhibition of tumor growth. These chemicals have demonstrated the capacity to modify cancer cell survival by modulating essential apoptotic pathways and oxidative indicators, hence substantiating the plant’s effectiveness against liver and breast cancer cells [45]. Specifically, gallic acid has been documented to downregulate anti-apoptotic proteins and upregulate pro-apoptotic factors in certain cell types, possibly contributing to noted cytotoxic effects [46].

Flavonoids such as rutin and quercetin, albeit in lesser amounts, augment the antioxidant and anticancer efficacy of the extract. These compounds exhibit anti-inflammatory and anti-metastatic effects, perhaps acting synergistically with other phenolics to diminish cancer cell proliferation and invasion. Quercetin is recognized for its ability to promote apoptosis and obstruct survival signaling pathways, hence augmenting the extract’s effectiveness [47].

Compounds with lower concentrations, such as caffeic acid and ferulic acid, contribute to the extract’s anticancer efficacy. These phenolics have been shown to impede cell proliferation by modulating oxidative stress and facilitating apoptosis, hence contributing to a cumulative anticancer effect even in minimal quantities [48,49].

The concurrent presence of these multiple compounds indicates a synergistic interaction potentially more effective in attacking cancer cells than any compound alone.

### 3.3 FTIR analysis

**Fig 2:**
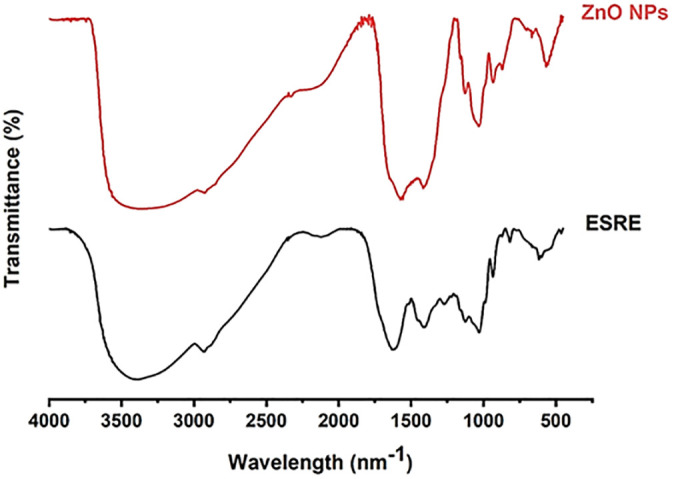
FTIR analysis of aqueous ESRE and green ZnO-NPs.

The ESRE extract and a representative sample of the ZnO-NPs underwent FT-IR analysis at ambient temperature to assess the functional groups of the plant metabolites involved in the reduction and stability of the nanoparticles Fig 2. The dual role of the plant extract, functioning as both a reducing agent and a capping agent, was corroborated through the FT-IR analysis of the synthesized ZnO-NPs. The FTIR spectrum of ESRE displayed significant peaks associated with essential functional groups that facilitate the reduction and stabilization of ZnO NPs.The broad peak at 3389 cm^-1^ corresponds to O–H stretching vibrations from phenolic and alcoholic groups, indicating high polyphenol content and flavonoid molecules [50]. The peak at 2933 cm^-1^ is attributed to C–H stretching of aliphatic chains, typical of fatty acids and terpenoids [51]. The minor peak at 2117 cm^-1^ may indicate the presence of alkynes or nitrile groups. A strong absorption at 1627 cm^-1^ is assigned to C = O or aromatic C = C stretching, consistent with flavonoids and phenolic acids [52]. The peaks at 1411 and 1273 cm^-1^ suggest O–H bending and C–O/C–N stretches, respectively, from phenolic and glycosidic compounds. Peaks at 1129 and 1031 cm^-1^ are typical for C-O stretching in alcohols and ethers, likely from sugars and glycosides [53]. Lower-frequency peaks at 935, 818, and 618 cm^-1^ represent aromatic ring deformations and out-of-plane C–H bending, confirming the presence of aromatic compounds. Altogether, the spectrum confirms a rich composition of phenolics, flavonoids, carbohydrates, and terpenoids in the root extract of *Echinops spinosus*. These bioactive chemicals, such as flavonoids, phenolics, tannins, and saponins are essential since they reduce zinc ions to ZnO NPs and offer a stabilizing layer on the nanoparticle surface during the synthesis of zinc oxide nanoparticles (ZnO NPs) from zinc acetate salt. [22]. These bio-components play a crucial role in controlling the size and shape of the nanoparticles and enhancing their surface stability, which directly affects the nanoparticles’ effectiveness in biological applications [54].

Flavonoids and phenolics contribute antioxidant properties and help minimize unwanted oxidation during synthesis [55]. Tannins and saponins improve nanoparticle dispersion and distribution, increasing surface interaction with target cells [56].

A comparative examination of the spectra derived from the aqueous ESRE and that of the extract incorporated in the synthesized ZnO-NPs revealed that upon the synthesis of ZnO NPs, spectral peak shifts and reduced band strength were observed, particularly in areas associated with hydroxyl and carbonyl groups. The O–H peak at around 3389 cm ⁻ ¹ demonstrated a little shift and diminished intensity, signifying interaction between hydroxyl groups in ESRE and Zn ions. The C = O stretching peak shifted from 1627 cm ⁻ ¹ to around 1580 cm ⁻ ¹, indicating the participation of carbonyl groups in nanoparticle production. These changes underscore the diminishing and limiting function of ESRE’s phytochemicals, as the hydroxyl and carbonyl groups are recognized for their ability to interact with metal ions during nanoparticle production [27].

The distinctive Zn–O stretching vibration was seen at around 500 cm ⁻ ¹, validating the synthesis of ZnO nanoparticles. The absence of this peak in the ESRE spectrum is distinctive to ZnO and constitutes compelling proof of the effective synthesis of ZnO nanoparticles. The existence of this peak indicates that Zn ions were successfully reduced and converted into ZnO by the ESRE components, aligning with prior research on the green synthesis of ZnO NPs [10,57–60]. The persistence of specific functional groups in the ZnO NP spectrum, though diminished in intensity, indicates the existence of residual plant chemicals on the nanoparticle surface. These remaining chemicals function as a natural capping layer, enhancing the dispersion stability and biocompatibility of the nanoparticles. The diminished strength in the O–H and C–O stretching bands signifies an effective capping effect, which is crucial for biomedical applications as it improves nanoparticle stability and mitigates toxicity to healthy cells [37].

### 3.4 Zeta potential analysis

**Fig 3:**
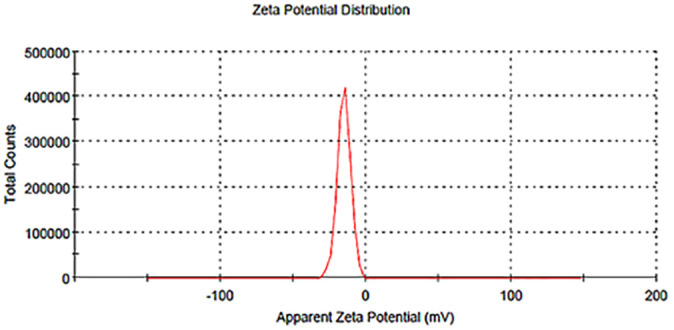
Zeta potential analysis of green ZnO-NPs.

The zeta potential of ZnO nanoparticles produced with ESRE extract was recorded at −15.2 mV, Fig 3, indicating moderate stability of the nanoparticles, suggesting a state of dispersion. This observation highlights the negative charge carried by the synthesized nanoparticles, with further implications of the involvement of phytochemicals from the plant extract in the reaction. The observed modest stability may be ascribed to the capping agents in the ESRE extract, which presumably enhance the negative surface charge. The components in the ESRE extract, including polyphenols, flavonoids, and other phytochemicals, may contribute to the zeta potential by adsorbing onto the surface of ZnO nanoparticles. These bioactive compounds are recognized for their capacity to adhere to metal oxide surfaces, thereby stabilizing nanoparticles and offering a natural, biocompatible capping layer. The bio-capping technique is beneficial since it improves stability and decreases toxicity, rendering the nanoparticles potentially appropriate for biomedical and environmental uses [61]. Zeta potential serves as a crucial measure of colloidal stability, as it signifies the surface charge and the likelihood of particle agglomeration. A zeta potential value exceeding ±30 mV is typically regarded as stable for nanoparticle suspensions, owing to robust electrostatic repulsion. Values ranging from −10 to −30 mV, including the observed −15.2 mV, suggest that the ZnO nanoparticles in this investigation may experience minor agglomeration over a long time, although being generally stable in their dispersion [62,63], a similar finding was stated for ZnO NPs from different plant extracts [63,64].

The obtained zeta potential indicates that the nanoparticles may exhibit diminished cellular toxicity relative to particles with greater negative or positive charges. Zeta potential affects both stability and interactions with biological systems; particles with near-neutral or slightly negative zeta potential are less likely to cause cellular toxicity or robust immune responses, which can be advantageous for certain applications in drug delivery or antimicrobial agents [65].

### 3.5 TEM analysis

**Fig 4:**
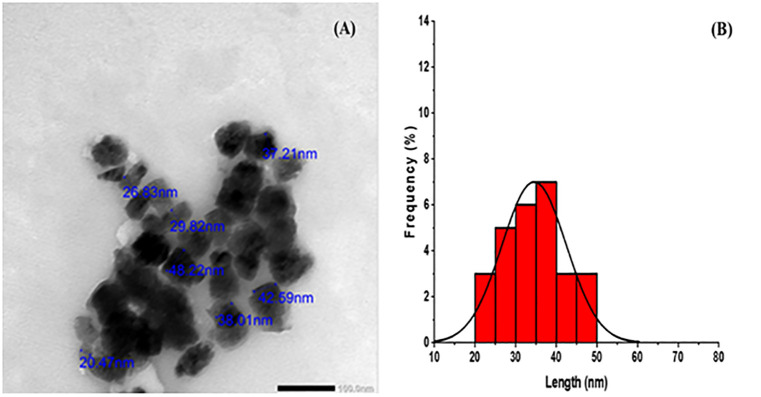
(A)TEM image and (B) histogram for TEM analysis of green ZnO-NPs.

The investigation utilizing transmission electron microscopy was performed to ascertain the morphology and average particle size of the ZnO nanoparticles produced via an ecologically friendly process. Some nanoparticles displayed transparency, whilst others showed thickness due to the overlap of nanoparticles. The images demonstrated that these nanoparticles were integrated inside an active template of organic compounds sourced from the aqueous (ESRE), suggesting their potential dual role in the reduction and capping processes during the synthesis of zinc oxide nanoparticles. Furthermore, the histogram analysis of the TEM image demonstrated that the nanoparticle particle sizes ranged from 20.47 to 48.22 nm, as shown in Fig 4. As a result, the ZnO nanoparticles displayed globular.

and hexagonal shapes with minor aggregations, a feature that aligns with prior research [66–68]. The values obtained from the test fall within the range reported by Amani et al., for the biosynthesis of zinc ferrite using plant extracts [20].

### 3.6 EDX analysis

**Fig 5:**
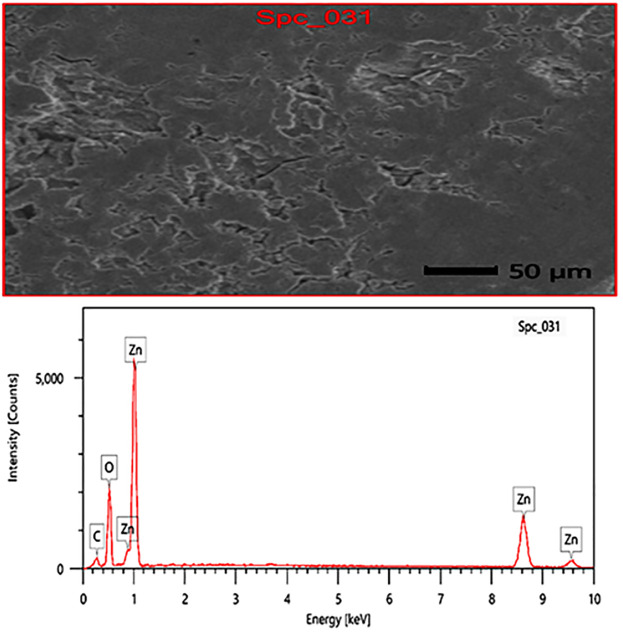
EDX analysis of green ZnO-NP.

The quantitative elemental composition of ZnO-NPs synthesized using green methods was examined through EDX analysis. The notable peaks identified in the EDX spectrum (Fig 5) at approximately 1 keV and 9 keV correspond to zinc (Zn) signals, signifying a substantial presence of Zn in the sample. The signal associated with oxygen at around 0.5 keV corroborates the production of ZnO, as oxygen is a fundamental constituent of the ZnO structure. The EDX measurement indicates that zinc comprises roughly [64.65%] of the sample, whereas oxygen constitutes around [26.31%]. A trace carbon (C) peak is observed in the low-energy region, presumably resulting from organic leftovers associated with the green synthesis method or sample preparation. This trace carbon may originate from plant extracts or natural compounds employed as capping and stabilizing agents, prevalent in green synthesis processes, and frequently imparts organic traces on the nanoparticles. The degradation of capping agents like polysaccharides, proteins, amino acids, and sugars because of X-ray emissions results in the emergence of the (C) peak [69].

The EDX results validate the effective synthesis of ZnO by utilizing the metabolites present in ESRE by verifying the elemental composition of Zn and O, consistent with ZnO nanoparticles. The elevated purity, evidenced by the lack of any notable elemental peaks, further substantiates the efficacy of green synthesis in generating ZnO nanoparticles with low contamination, as recorded in analogous investigations on the green synthesis of ZnO [37,70–73].

### 3.7 Biological evaluation

The ESRE and green synthesized ZnO NPs were estimated for their *in vitro* anticancer activities *via* the standard MTT method [74–78], against a panel of two human cancer cell lines: breast cancer (MCF-7) and hepatocellular carcinoma (HepG2). Doxorubicin (DOX) was utilized as a positive control. The results were expressed for each compound as half maximal inhibitory concentration (IC_50_) values.

**Fig 6:**
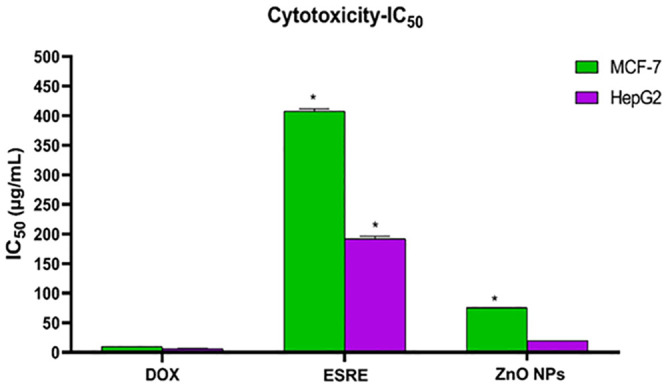
In vitro cytotoxic activities of the ESRE and ZnO NPs against MCF-7 and HepG2 cell lines. *: Significant from DOX group at *p* < 0.001.

ZnO NPs exhibited significantly superior anti-proliferative activity, being approximately 10.4 folds more effective in HepG2 cells and 5.4 folds more effective in MCF-7 cells compared to ESRE. ZnO NPs exhibited IC50 values of 19.94 µg/mL for HepG2 and 75.65 µg/mL for MCF-7, whereas ESRE demonstrated IC50 values of 191.8 µg/mL for HepG2 and 407.69 µg/mL for MCF-7, signifying substantially lower potency (Table 2, Fig 6).

**Table 2:** In vitro cytotoxic activities of the ESRE and ZnO NPs against MCF-7 and HepG2 cell lines.

Comp. ID	In vitro cytotoxicity IC_50_ (µg/mL)	
	MCF-7	HepG2
**Doxorubicin (DOX)**	9.65 ± 0.19	6.68 ± 0.17
**ESRE**	407.69 ± 3.71	191.8 ± 4.43
**ZnO NPs**	75.65 ± 0.16	19.94 ± 0.11

Results were expressed as mean ± SD.

Despite ZnO NPs being less potent than DOX, they attained 33% of DOX’s efficacy against HepG2 cells and 13% against MCF-7 cells. DOX, serving as a standard control, exhibited IC50 values of 6.68 µM for HepG2 and 9.65 µM for MCF-7, highlighting its significant potency.

HepG2 cells exhibited greater sensitivity to both ZnO nanoparticles and ESRE compared to MCF-7 cells. This differential response indicates that ZnO NPs may employ selective cytotoxic mechanisms that are more efficacious in hepatocellular cancer cells. Variations in sensitivity may arise from variances in cellular absorption, inherent cellular resistance, or distinct oxidative stress responses among cell types. HepG2 cells exhibit greater sensitivity to oxidative stress than other cell lines, attributable to their elevated metabolic activity and the liver’s inherent susceptibility to ROS-induced toxicity. This reactive oxygen species generation impairs mitochondrial function, facilitating caspase activation and apoptotic pathways more effectively in HepG2 cells compared to MCF-7 cells [79,80]. In addition, HepG2 cells may demonstrate increased uptake of ZnO nanoparticles owing to their membrane properties, which promote enhanced nanoparticle infiltration. Research demonstrates that liver cancer cells exhibit a greater propensity to accumulate nanoparticles compared to breast cancer cells, resulting in elevated intracellular ZnO NP concentrations in HepG2 cells and, subsequently, more significant cytotoxic effects. The enhanced absorption is essential for eliciting cytotoxicity, as ZnO nanoparticles interact more efficiently within the cells, resulting in elevated cell mortality [81].

Our IC₅₀ values for ZnO NPs were 19.94 µg/mL for HepG2 and 75.65 µg/mL for MCF-7 cells, which compare favorably to those reported by Zahra et al., who synthesized ZnO NPs using *Sargassum muticum* algae extract and observed IC₅₀ values of 150 μg/mL at 48 hours of exposure against HepG2 cells [82]. Similarly, Amir et al. reported an IC₅₀ of 11.16 μg/mL against MCF-7 cells using ZnO NPs synthesized from *Saponaria officinalis*, which is comparable to our result but with a higher effective dose [83]. Ruqaya et al. found IC₅₀ values around 14.7 μg/mL for breast cancer cell lines using *Zingiber officinale*-mediated ZnO NPs, slightly more potent than our ESRE-mediated particles for MCF-7 [84], possibly due to differing phytochemical content and particle morphology, indicating our particles are more cytotoxic in hepatic models. These comparisons suggest that *Echinops spinosus*-based ZnO NPs demonstrate competitive or superior anticancer efficacy, particularly for liver cancer applications, justifying their further development. Thanks to the interactive relationship between the bio-components and ZnO nanoparticles, the physicochemical properties of the particles were improved, enabling them to effectively affect cancer cells in our experiment [85,86].

**Fig 7:**
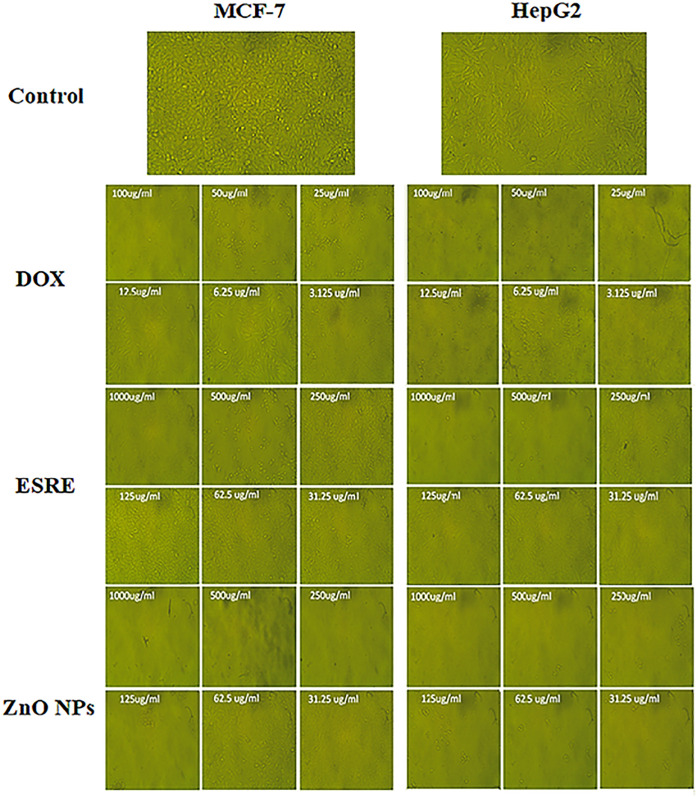
Effect of DOX, ESRE, and ZnO NPs on MCF-7 and HepG2 cells at different concentrations.

Fig 7 depicts the dose-dependent cytotoxicity of DOX, ESRE, and ZnO NPs at escalating concentrations. The significant reduction in cell viability at elevated doses of ZnO NPs and DOX, especially in HepG2 cells, underscores the efficacy of these agents in suppressing cancer cell proliferation. The dose-response curve demonstrates that HepG2 cells exhibit greater sensitivity to ZnO nanoparticles than MCF-7 cells, validating the computed IC50 values.

The enhanced anticancer efficacy of ZnO NPs is connected with their capacity to produce reactive oxygen species (ROS), which provoke oxidative stress and apoptosis in neoplastic cells. ZnO NPs compromise mitochondrial membrane integrity, resulting in death via caspase activation [87]. Moreover, ZnO NPs selectively accumulate in cancer cells, potentially minimizing the impact on normal cells. This selective cytotoxicity is beneficial for therapeutic applications since it may mitigate the negative effects linked to non-specific cytotoxic drugs.

Although ZnO NPs exhibited reduced cytotoxicity compared to DOX, they represent a promising alternative, particularly due to their selective accumulation in cancer cells and potential for minimal effects on normal cells. The green synthesis method for ZnO nanoparticles enhances their feasibility as a more sustainable and perhaps safer anticancer drug than traditional chemical production techniques. Conversely, ESRE’s elevated IC50 values indicate its restricted effectiveness as an independent anticancer agent, however, it may provide supplementary advantages when used in conjunction with ZnO NPs. Moreover, the unique biological and physicochemical properties of the green-synthesized ZnO nanoparticles can be largely attributed to the interactive role of bioactive compounds present in *Echinops spinosus* root extract. Phenolics, flavonoids, saponins, and tannins act as natural reducing and stabilizing agents during nanoparticle synthesis. Hydroxyl and carbonyl groups from these compounds chelate zinc ions and mediate their conversion into nanoscale ZnO structures, while simultaneously capping the nanoparticle surface. This capping layer enhances colloidal stability, reduces aggregation, and contributes to biocompatibility. Residual phytochemicals adhered to the ZnO NP surfaces may modulate biological interactions, such as cellular uptake and oxidative stress induction, thus influencing their anticancer potential. The synergistic effect between ZnO core properties and surface-bound bio-components underlies the observed selective cytotoxicity, particularly toward HepG2 cells, and supports the multifunctional application of these NPs in cancer nanomedicine.

Previous studies have shown that ZnO NPs can accumulate in various organs, such as the liver, spleen, and lungs, due to their nanoscale size and ability to exploit the enhanced permeability and retention (EPR) effect [88,89]. Additionally, ZnO NPs undergo partial dissolution in biological fluids, releasing Zn² ⁺ ions and interacting with biological macromolecules, leading to biotransformation processes such as protein corona formation and enzymatic modification [90]. While green-synthesized zinc oxide nanoparticles (ZnO NPs) offer advantages such as eco-friendly production, biocompatibility, and anticancer potential, they still face important limitations. One major concern is that the biological variability in plant extracts or microbial sources leads to inconsistent NP size, morphology, and surface properties, affecting therapeutic efficacy, biodistribution, and cellular uptake [91]. Additionally, green methods face low yields compared to chemical synthesis, hindering mass production [84]. Biological processes also lack standardized protocols for large-scale manufacturing [92]. Furthermore, long-term exposure, clearance mechanisms, and accumulation in organs like the liver and spleen remain incompletely understood, especially for repeated or chronic exposure scenarios [88,90]. However, these limitations can be overcomed by using genetically engineered microbes or standardized plant extracts to reduce batch variability and use plant extracts for nucleation, followed by controlled chemical growth to boost yield while retaining biocompatibility.

## 4. Conclusion

This study successfully demonstrated the green synthesis of zinc oxide nanoparticles (ZnO-NPs) using *Echinops spinosus* root extract, a medicinal plant not previously reported for this purpose. The resulting ZnO-NPs exhibited well-defined physicochemical characteristics and significant in vitro anticancer activity. Specifically, the ZnO-NPs achieved an IC₅₀ value of 19.94 ± 0.11 µg/mL against HepG2 liver cancer cells and 75.65 ± 0.16 µg/mL against MCF-7 breast cancer cells, indicating a stronger and more selective cytotoxic effect toward hepatocellular carcinoma. Compared to other studies using plant-mediated ZnO NPs, our nanoparticles demonstrated comparable or superior efficacy, highlighting the potency of *Echinops spinosus*-derived phytochemicals as reducing and capping agents.

The novelty of this work lies in the use of *Echinops spinosus* for both nanoparticle synthesis and biological evaluation, supported by phytochemical profiling, zeta potential analysis, and transmission electron microscopy. The integration of green chemistry with targeted anticancer screening supports the feasibility of using such biosynthesized ZnO-NPs in early-stage therapeutic development. These findings underscore the promise of green nanotechnology in developing sustainable and potent anticancer therapies.

## Supporting information

S1 File Inclusivity in global research. (DOCX)
