# Supplementary material for: Green-synthesized zinc oxide nanoparticles using Echinops spinosus extract: A promising anticancer agent for hepatocellular carcinoma
Source: PLoS One. 2025 Oct 23;20(10):e0331171. doi: 10.1371/journal.pone.0331171 (PMC12548911; doi:10.1371/journal.pone.0331171)
Supplement: S1 File — (DOCX) [file pone.0331171.s001.docx]

Inclusivity in global research

PLOS’ policy on inclusivity in global research aims to improve transparency in the reporting of research performed outside of researchers’ own country or community and ensures that PLOS publications reporting global research adhere to high standards for research ethics and authorship. Authors of relevant research articles may be asked to complete the questionnaire below, which outlines ethical, cultural, and scientific considerations specific to inclusivity in global research. This questionnaire may be requested when researchers have travelled to a different country to conduct research, if research uses samples collected in another country, research with Indigenous populations or their lands, or if research is on cultural artefacts. Researchers travelling to another country solely to use laboratory equipment will not normally be required to complete the questionnaire. However, the questionnaire can be requested at the journal’s discretion for any submission – if you have been requested to complete this questionnaire by the PLOS journal you submitted to, please do so.

Please complete the questionnaire below and include this as a Supporting Information file with your manuscript. Note that if your paper is accepted for publication, this checklist will be published with your article in the supporting information files. Please ensure that you reference the checklist in the main body of your manuscript. We suggest adding a subsection ‘Inclusivity in global research’ to your Methods section and adding the following sentence: “Additional information regarding the ethical, cultural, and scientific considerations specific to inclusivity in global research is included in the Supporting Information (SX Checklist)”

The questions have been designed to be applicable to a wide range of study types, and there are subsections for both human subjects research and non-human subjects research. If any of the questions are not relevant to your research please mark them as “N/A” as appropriate.

**Ethical considerations, permits and authorship**

*This section is applicable to all research types.*

Provide details as to who granted permissions and/or consent for the study to take place in the Methods section of your manuscript. This should include the names of **all** ethics boards, governmental organizations, community leaders or other bodies that provided approval for the study. If individuals provided approval refer to these people by their role or title but do not list their name(s).

This study didn’t involve any animal or human experiments. The BUC University Research Ethics Committee has confirmed that no ethical approval is required, as only established cell lines were used and no human or animal subjects were directly involved (Page No. 24).

If there were any deviations from the study protocol after approval was obtained please provide details of these changes in the Methods section of your manuscript.

There is no indication or report of any deviations from the approved study protocol

Did this study involve local collaborators that are residents of the country where the research was conducted or members of the community studied? If you do not have any authors from said communities, please provide an explanation for this below.

*This study did not include local collaborators who are residents of the country where the research was conducted or members of the community studied. The research team was composed of individuals with expertise in nanotechnology, phytochemistry, and cancer biology, and all plant materials were collected in accordance with national guidelines. No direct community engagement or local authorship was established for this project due to the nature of the research and the use of commercially available cell lines*

Everyone listed as an author should meet PLOS’ criteria for authorship and all individuals who meet these criteria should be included in the author byline, rather than the acknowledgements. For further information please see the journal’s Authorship Policy.

**Human subjects research (e.g. health research, medical research, cross-cultural psychology)**

Did you obtain written informed consent from a representative of the local community or region before the research took place? How did you establish who speaks for the community? Details of written informed consent obtained from study participants should be reported separately in the Methods section of your manuscript.

No human participants or animals were involved in this study. All cell lines used were obtained from certified repositories (ATCC), and their use complied with institutional and national ethical guidelines. No informed consent was required

How did members of the local community provide input on the aims of the research investigation, its methodology, and its anticipated outcome(s)?

there is no members of the local community provided input on the aims, methodology, or anticipated outcomes of the research

When engaging with the local community, how did you ensure that the informed consent documents and other materials could be understood by local stakeholders?

Will the findings of the research be made available in an understandable format to stakeholders in the community where the study was conducted (e.g. via a presentation, summary report, copies of publications, etc.)? Please provide details of how this will be achieved.

No, this study did not involve direct engagement with local community members, nor did it require informed consent documents for community stakeholders.

**Non-human subjects research using specimens/ animals collected as part of the study, or those housed in archival collections. Examples include archaeology, paleontology, botany and zoology.**

Did the permission you obtained from a local authority to perform the study include an agreement on access to outputs and benefit sharing? This may include procedures to enable fair distribution of the benefits and resources arising from the research performed. Please include any details of Prior Informed Consent and Benefit Sharing Agreements obtained. These may be required by field-specific regulations, for example the Convention on Biological Diversity (CBD) and the associated Nagoya Protocol.

This research did not include a formal agreement regarding access to research outputs or benefit sharing with local authorities or the community.

If the material used in your study was imported, please A) provide the year it was imported and B) indicate whether permits were obtained to import/export the materials used, C) provide details of any permits obtained. If this information is not available, please indicate this.

All plant materials (Echinops spinosus roots) used in this study were collected locally in Egypt and were not imported; therefore, no import or export permits were required.

If you used archival specimens, please state how the material used in your study was acquired by the institute it is held in and provide details of any permits obtained for the original excavations/ sample collection. If this information is not available, please indicate this.

No, archival specimens were not used in this study.

How was the potential cultural significance of the materials collected in your study to local communities considered in your research design? Were Indigenous peoples and/or local researchers and institutions involved with archaeological excavations / collection of specimens? If so, please provide a description of their involvement.

The potential cultural significance of Echinops spinosus to local communities was not formally assessed in the research design. The plant material was collected from a publicly accessible area near Borg El Arab, Alexandria, Egypt, for scientific investigation of its properties in green nanoparticle synthesis and anticancer activity. No Indigenous peoples or local community representatives were involved in the collection of specimens or in the design or execution of the study. If future research involves resources of significant cultural or traditional importance, the research team will seek to engage with relevant communities and authorities to ensure ethical and culturally sensitive research practices

If your manuscript includes photographs of human remains please indicate whether authors obtained permission from descendants or affiliated cultural communities to do so.

This manuscript does not include photographs of human remains. Therefore, obtaining permission from descendants or affiliated cultural communities was not applicable.
